# Former smoking as a risk factor for visual field progression in exfoliation glaucoma patients in Sweden

**DOI:** 10.1177/11206721241226990

**Published:** 2024-01-17

**Authors:** Marcelo Ayala

**Affiliations:** Eye Department, Skaraborg Hospital, Sahlgrenska Academy, Gothenburg University & Karolinska Institute, Skövde, Sweden

**Keywords:** Exfoliation glaucoma, visual field progression, risk factors, smoking, Sweden.

## Abstract

**Purpose:**

The present study aimed to identify whether former smoking was a risk factor for visual field progression in exfoliation glaucoma patients.

**Methods:**

Prospective nonrandomised cohort study. The study included patients diagnosed with exfoliation glaucoma. All included patients were followed for three years (± three months) with reliable visual fields. At least five reliable visual fields needed to be included in the study. Exfoliation glaucoma was defined using the European Glaucoma Society Guidelines. The visual fields were tested using the 24–2 test strategy of the Humphrey Field Analyzer. Smoking was assessed through questionnaires. Outcomes: Visual field progression. Three different approaches were used: difference in mean deviation (MD), rate of progression (ROP), and guided progression analysis (GPA).

**Results:**

In total, *n* = 113 patients were included; among them, *n* = 57 were smokers. Smoking was a significant predictor for visual field progression in the three models (MD/ROP/GPA) studied (*p* = 0.01/*p* = 0.001/p ≤ 0.001), even adjusting for intraocular pressure (IOP). Other predictors were included in the MD model: IOP at diagnosis (*p* = 0.04) and selective laser trabeculoplasty (SLT) treatment (*p* = 0.01). Other predictors were in the ROP model: Visual field index (*p* = 0.005), number of medications (*p* = 0.001) and SLT treatment (*p* = 0.001). The number of medications was another predictor in the GPA model (*p* = 0.002).

**Conclusions:**

Former smoking induced visual field deterioration in all models studied. Smoking status should be considered when establishing the glaucoma diagnosis. Increased glaucoma care should be provided to former smokers to slow the progression of the disease.

## Background

Glaucoma is a chronic, irreversible, potentially blinding eye disease that affects the optic nerve.^1^ Usually, visual field defects develop in glaucoma; in advanced cases, glaucoma can even lead to blindness.^2^ Primary open-angle glaucoma (POAG) and secondary exfoliation glaucoma (EXFG) are the two most common clinical manifestations of glaucoma in Sweden.^3^ Exfoliation glaucoma is due to the accumulation of a greyish protein-based material in the eye's anterior chamber. Exfoliation material has been identified in different eye structures, but even extraocular organs reveal the presence of exfoliation material. Exfoliation material has been isolated in the lung, heart, liver, and gallbladder, in addition to the classic intraocular sites.^4–7^

Risk factors for the onset and progression of glaucoma have been well described in previous studies.^8–10^ In the Early Manifest Glaucoma Trial (EMGT),^11^ the risk factors for glaucoma progression were identified as higher intraocular pressure (IOP), exfoliation, increased damage at baseline, higher age, and disc haemorrhages. The EMGT studied glaucoma progression based on mean deviation (MD) values. Other studies confirmed that age and IOP were significant risk factors for glaucoma progression in POAG patients.^12–16^ Very little evidence about specific risk factors for visual field deterioration in EXFG was found.

Smoking has been extensively described as a risk factor for health. Smoking has been identified as a significant risk factor for cardiovascular disease, carotid disease, and peripheral artery disease.^17^ In the eye, the association between smoking and glaucoma has been described in previous studies with different results.^18–27^ Four systematic reviews showing different results were found in the literature.^28–31^ Edwards, R. et al. showed a weak association between smoking and glaucoma; the review did not consider the smoking status (current or former). Jain V. et al. showed more robust evidence of an association between smoking and glaucoma among current smokers than among former smokers. Bonovas S. et al. showed an increased risk with current smoking; the pooled estimate OR was 1.37, but no risk was found in the case of former smokers. However, Zhou, Y. et al. showed no association between smoking and glaucoma, both in current and former smokers (pooled RR 0.97 in both). The results were variable due to differences in the studies, including different populations, study designs, and how smoking was assessed and classified. All of the studies in the aforementioned systematic reviews ranked glaucoma as a binary variable (Yes/No), included only POAG patients, and did not consider the disease stage and/or progression.

The present study aimed to identify whether former smoking was a risk factor for visual field progression in EXFG.

## Methods

The present study was a prospective nonrandomised cohort study. The study followed the STROBE guidelines.^32^ All patients diagnosed with exfoliation glaucoma attending the Ophthalmology Department at Skaraborg's Hospital were asked to participate in the study. The recruiting period was from January 2014 until December 2018 (five years). The study adhered to the tenets of the Declaration of Helsinki. All patients signed informed consent forms. The University of Gothenburg granted ethical approval (DN: 119-12).

To be eligible for the study, patients had to be diagnosed with exfoliation glaucoma, characterised by exfoliation material in the anterior chamber and considered a secondary open-angle glaucoma, according to the European Glaucoma Society. Additionally, patients had to be 85 years old or younger at the time of recruitment.

To ensure the accuracy of our study, we established exclusion criteria for participants. Those who could not perform at least five reliable visual fields three years after recruitment were excluded. Reliable visual fields were determined by false positives of 15% or less, false negatives of 20% or less, and fixation losses of 30% or less. We also excluded patients with advanced visual field deterioration, defined as a mean deviation (MD) of −18 dB or more and/or a visual field index (VFI) of 40% or less to avoid “floor effects”. We excluded patients who underwent glaucoma surgery during the follow-up period, those with other eye diseases that could modify visual fields, and those who dropped out of the control period. Finally, we also excluded patients who took nicotine in forms other than cigarette smoking.

The risk factors were recorded at three stages: glaucoma diagnosis, recruitment and after the three-year (± three months) follow-up period. At diagnosis, the risk factors recorded were age and IOP. At the recruiting visit, the following risk factors were recorded: smoking, age, sex, unilateral/bilateral glaucoma, visual acuity, refractive errors, IOP, number of medications, central corneal thickness (CCT) measurement, gonioscopy evaluation (depth and pigmentation), cup-disc (C/D) ratio, diabetes, hypertension, cardiovascular diseases (including angina, heart attacks, heart failure, stroke, TIA and peripheral arterial diseases), migraine and family history of glaucoma. The risk factors studied during the three years were IOP reduction, SLT treatment, cataract operation, and the number of medications used.

At the recruiting visit, all participants completed a questionnaire regarding smoking (Supplemental material). Smoking was studied and classified as “not a smoker/former/current”, according to the definitions of the Centers for Disease Control and Prevention (CDC) National Health Interview Services.^33^

Furthermore, the questionnaire included questions regarding hypertension, cardiovascular diseases, diabetes, migraine, and a family history of glaucoma.

Every patient was ophthalmologically examined by the same ophthalmologist (MA) at the recruitment visit. If both eyes were suffering from glaucoma, both eyes were registered, but only one eye, chosen at random (sealed envelopes), was included in the study.

The risk factors for progression under the three-year follow-up were measured: IOP reduction was calculated as the difference in IOP between the IOP at diagnosis and the IOP three years after recruitment. The IOP was checked every fourth or fifth month. The IOP was measured using a Goldman's tonometer; IOP was measured three times, and an average value was calculated. The SLT treatment was measured as present or absent per patient (yes/no). Cataract operation was counted as present or absent (yes/no). Eye drops at the end of the three years were measured as the number of medications (compounds).

### Endpoints

The primary endpoint of the study was progression of the visual field. All patients were examined using the Humphrey Field Analyzer device (Carl Zeiss, Carl-Zeiss-Straße 22, 73447 Oberkochen, Germany) and the test point pattern 24–2. Visual field progression was assessed in three different ways.

The first approach was based on the mean deviation (MD) values. The difference in MD values was calculated from the beginning to the end of the study. The MD parameter was chosen as several studies still use MD as an indicator of progression.^34–37^

The second method used to analyse progression was based on the visual field index (VFI) values. The machine calculated the VFI and performed a regression analysis calculating the rate of progression (ROP).

Finally, guided progression analysis (GPA) was the third evaluation method. GPA is included in the device and performed automatically (GPA Alert). GPA Alert showed no, possible or likely progression. The present study evaluated the results as “no progression” or “progression”. The term “progression” included both “possible” and “likely” progression.

Swedish ophthalmologists use both the VFI and GPA analysis in daily practice to determine visual field deterioration in glaucoma.

### Statistics

SPSS statistical software was used for statistical analysis (IBM, 1 New Orchard Road Armonk, NY 10504, USA). All of the studied variables were tested in two steps. The first step used univariate linear regression analysis for continuous endpoint variables (MD and ROP). Meanwhile, the dichotomous endpoint (GPA) used a univariate logistic regression. The variables that showed significant values in the univariate analysis model were included as covariates in a multivariate analysis. Multivariate linear regression analysis was used in case the endpoints were continuous (MD and ROP). Multivariate logistic regression analysis was performed in the case of a dichotomous variable (GPA). In addition, a subgroup analysis among the smokers using a similar strategy was performed. The significance level was set at 0.05. A power analysis for MD values based on an unpaired T-test was done. The power analysis showed that a sample size of at least 24 experimental subjects was needed to detect a difference of 2.5 dB in MD in 3 years with a standard deviation of 3dB, a power of 0.80, and a significance level of 0.05.

## Results

In total, *n* = 113 patients were included in this cohort study. The cohort was studied according to smoking status. Glaucoma diagnosis was presented more often bilaterally among smokers than among nonsmokers (chi-square: *p* = 0.03). The visual field parameters (MD and VFI) at recruitment did not differ between smokers and nonsmokers. See Table 1.

**Table 1.** Baseline clinical characteristics of the patients according to smoking status.

	No smokers *N* = 56	Smokers *N* = 57	Test	P-value
Sex (M/F) (%)	24/32 (43/57)	28/29 (49/51)	Chi-square	0.45
Age at diagnosis (years) (SD)	70.35 (±6.43)	69.78 (±5.45)	T-test	0.18
Age at recruitment (years) (SD)	72.34 (±7.34)	71.07 (±6.67)	T-test	0.23
IOP at diagnosis (mmHg) (SD)	32.96 (±6.37)	33.72 (±6.55)	T-test	0.07
IOP at recruitment (mmHg) (SD)	17.96 (±2.47)	17.63 (±3.09)	T-test	0.89
Fakia/pseudofakia at inclusion (%)	40/16 (71/29)	42/15 (74/26)	Chi-square	0.76
Unilateral/bilateral (%)	45/11 (80/20)	36/21 (63/37)	Chi-square	0.03*
CCT (µm) (SD)	542.44 (±36.71)	544.72 (±30.95)	T-test	0.72
Number of medicines at recruitment	1.7 (±0.74)	1.87 (±0.79)	T-test	0.20
Visual field MD at recruitment (dB) (SD)	−5.59 (±4.57)	−6.72 (±5.19)	T-test	0.06
Visual field VFI at recruitment (%) (SD)	87.83 (±12.23)	85.06 (±15.07)	T-test	0.07

M: Male. F: Female. SD: Standard deviation. IOP: Intraocular pressure. CCT: Central cornea thickness. MD: Mean deviation. VFI: Visual field index.

(*) Significant values

During the three years of follow-up, the number of patients treated with SLT was significantly higher among smokers than nonsmokers (chi-square: *p* = 0.005). At the end of the three-year follow-up period, the MD and the VFI values differed between the smokers and the nonsmokers. The smokers showed a more damaged visual field than the nonsmokers (T-test: *p* = 0.002 for MD and *p* = 0.003 for VFI). In addition, the progression parameters (MD/ROP and GPA) differed between smokers and nonsmokers after three years of follow-up (T-test: p ≤ 0.001/*p* = 0.001/chi-square: p ≤ 0.001). See Table 2.

**Table 2.** General clinical characteristics of the patients after three years’ follow-up according to smoking status.

	No smokers *N* = 56	Smokers *N* = 57	Test	P-value
Cataract operation during the 3 years (%)	9/47 (16/84)	6/51 (11/89)	Chi-square	0.57
IOP at 3 years (mmHg) (SD)	16.88 (±2.81)	16.82 (±2.95)	T-test	0.92
Number of medicines at 3 years (SD)	2.55 (±0.93)	2.89 (±0.74)	T-test	0.06
SLT treated/untreated (%)	6/50 (11/89)	17/40 (30/70)	Chi-square	0.005*
Visual field MD at 3 years (dB) (SD)	−8.79 (±5.26)	−11.72 (±6.69)	T-test	0.002*
Visual field VFI at 3 years (%) (SD)	79.71 (±16.20)	69.03 (±20.16)	T-test	0.003*
MD difference at 3 years (dB) (SD)	3.20 (±1.85)	5.01 (±2.86)	T-test	<0.001*
ROP during 3 years (%/year) (SD)	−1.63 (±1.71)	−3.83 (±2.31)	T-test	0.001*
GPA (no progress/progress) (%)	32/24 (56/44)	10/47 (18/82)	Chi-square	<0.001*

IOP: Intraocular pressure. MD: Mean deviation. VFI: Visual field index. SLT: Selective laser trabeculoplasty. ROP: rate of progression.

(*) Significant values

The independent variables were first tested in a univariate regression model for the dependent variables: MD, ROP, and GPA. Only variables showing a significant value (p ≤ 0.05) were included in a multivariable analysis. In the multivariable linear regression MD model, the variables that showed a significant association were smoking (*p* = 0.01), IOP at diagnosis (*p* = 0.04), and SLT treatment (*p* = 0.01). Please see Table 3.

**Table 3.** Significant variables were tested in the univariate and multivariate linear regression analysis using the MD difference as the endpoint.

Variables	Univariate		Multivariate	
	β coeff. (SE)	P-values	β coeff. (SE)	P-values
Smoking	0.51 (0.42)	<0.001*	0.37 (0.43)	0.01*
Age at diagnosis	0.04 (0.02)	0.02*	0.06 (0.03)	0.34
IOP at diagnosis	0.43 (0.45)	0.01*	0.19 (0.04)	0.04*
MD at recruitment	0.34 (0.05)	0.001*	0.11 (0.17)	0.71
VFI at recruitment	0.31 (0.01)	0.001*	0.04 (0.05)	0.89
Number of medications at 3 years	0.35 (0.26)	<0.001*	0.16 (0.24)	0.06
SLT treatment	0.39 (0.27)	<0.001*	0.20 (0.34)	0.01*

β coeff.: β coefficient. SE: Standard error. IOP: Intraocular pressure. MD: Mean deviation. VFI: Visual field index. SLT: Selective Laser Trabeculoplasty.

(*) Significant values

In the multivariate linear regression using ROP as the endpoint, the variables associated with progression were smoking (*p* = 0.001), VFI at recruitment (*p* = 0.005), the number of medications at the three-year follow-up (*p* = 0.001) and SLT treatment (*p* = 0.001). Please see Table 4.

**Table 4.** Significant variables were tested in the univariate and the multivariate linear regression analysis using the ROP as the endpoint.

Variables	Univariate		Multivariate	
	β coeff. (SE)	P-values	β coeff. (SE)	P-values
Smoking	0.48 (0.38)	<0.001*	0.29 (0.26)	0.001*
Age	0.04 (0.02)	0.03*	0.06 (0.04)	0.08
IOP at 3 years	0.43 (0.03)	0.001*	0.01(0.02)	0.10
MD at recruitment	0.50 (0.04)	0.001*	0.82 (0.10)	0.07
VFI at recruitment	0.43 (0.01)	<0.001*	0.63 (0.04)	0.005*
Number of medications at 3 years	−0.52 (0.19)	<0.001*	−0.28 (0.15)	0.001*
SLT	−0.59 (0.43)	<0.001*	−0.36 (0.33)	0.001*

β coeff.: β coefficient. SE: Standard error. IOP: Intraocular pressure. MD: Mean deviation. ROP: Rate of progression. SLT: Selective Laser Treatment.

(*) Significant values.

In the multivariate logistic regression GPA model, the variables associated with progress were smoking (p ≤ 0.001) and the number of medications at the three-year follow-up (*p* = 0.002). Age and SLT treatment showed no significance in the univariate analysis. Please see Table 5.

**Table 5.** Significant variables were tested in the univariate and multivariate logistic regression analysis using the endpoint of the GPA (dichotomous: progress/no progress).

Variable	Univariate		Multivariate	
	P-values	OR (95% CI)	P-values	OR (95% CI)
Smoking	<0.001*	5.67 (2.45–13.23)	<0.001*	8.77 (2.84–34.78)
IOP at diagnosis	0.001*	1.15 (1.07–1.25)	0.49	0.96 (0.86–1.08)
Number of medications at 3 years	<0.001*	3.23 (1.25–5.23)	0.002*	3.65 (1.57–7.67)
MD at recruitment	<0.001*	0.78 (0.69–0.87)	0.06	0.62 (0.38–1.02)
VFI at recruitment	0.001*	0.92 (0.88–0.96)	0.26	1.09 (0.93–1.29)

GPA: Guided progression analysis. OR: Odds ratio. IOP: Intraocular pressure. MD: Mean deviation. VFI: Visual field index.

(*) Significant values.

### Subgroup analysis

The smoking patients were further analysed. Most included patients were “former smokers” (*n* = 54/95%). The average age at recruitment was 72.27 (±7.24) years. The sex distribution was *n* = 28 males and *n* = 29 females. The average age at which they began smoking was 17.08 (±2.62) years. The average age at which they quit smoking was 45.17 (±13.97) years. The patients’ average smoking time was 29.08 (±13.77) years. The average number of cigarettes per day they smoked was *n* = 10.13 (±4.16). Only two participants smoked ≥ 20 cigarettes/day (heavy smokers). The multivariate regression analysis showed an association between the number of cigarettes and visual field deterioration in the three models studied (MD model: *p* = 0.005, ROP model: p ≤ 0.001, and GPA model: *p* = 0.03). In the multivariate analysis, the model was adjusted for the number of medicines, SLT treatment, IOP at diagnosis, MD, and VFI at recruitment. Please see Table 6.

**Table 6.** Subgroup analysis testing the association between the number of cigarettes smoked during life and visual field deterioration in the three models studied.

Model	Univariate		Multivariate ^(1)^	
	β coeff.	P-values	β coeff.	P-values
MD	0.35	0.008*	0.31	0.005*
ROP	0.36	0.006*	0.27	<0.001*
GPA	1.01^(2)^	0.04*	1.02^(2)^	0.03*

1) Adjusted for the number of medicines, SLT treatment, IOP at diagnosis, and VFI at recruitment.

β coeff.: β coefficient. MD: Mean deviation. ROP: Rate of progression. GPA: Guided progression analysis.

(*) Significant values. (2) “Exp B” (OR) in the logistic regression.

## Discussion

The present study showed an association between former smoking and visual field deterioration in EXFG patients in Sweden. The association was presented in the three glaucoma progression models studied (MD, ROP and GPA). Even after adjusting for the most common progression factor (IOP), smoking remained a predictor.

The bilateral presentation of glaucoma was more common among smokers than nonsmokers. Some kinds of systemic factors induced by nicotine use could explain why glaucoma was more often bilateral among smokers than among nonsmokers. At baseline, no difference was found between smokers and nonsmokers regarding visual field parameters. However, after a three-year follow-up period, smokers showed increased progress in their visual field damage compared with nonsmokers. The three models used (i.e., MD difference/ROP/GPA) showed significant results. In the MD model, the difference between the MD values from the beginning to the end of the study was 2 dB among nonsmokers and approximately 5 dB among smokers. Similar results were shown in the ROP model; the progression among smokers (−3.83%/year) was more than double that among nonsmokers (−1.63%/year). The GPA model also showed that 44% were classified as progression in the nonsmoker group, while 82% were classified as progression in the smoker group.

According to several previous studies, the IOP is the most critical factor for predicting VF deterioration.^12–16^ In the present study, IOP was studied at three different time points: at diagnosis, recruitment and three-year follow-up. The decision to include only three different time points was made because many IOP measurements would not add more information to the study. The IOP at diagnosis was significantly associated with visual field progression in the MD model but not in the other models. The IOP at three years was significant in the univariate model considering the ROP model; however, it failed to show significance when the variable was included in the multivariable ROP model. Treatment for IOP reduction was performed on a clinical basis. Although there was a reduced IOP in both smokers and nonsmokers, IOP reduction was not as effective in stopping progression among smokers. There are two different possible explanations: 1) smokers need a lower IOP to stop progression, and 2) visual field progression among smokers is not related to IOP. Furthermore, a combination of both factors is also possible. It is wise to often recommend visual field tests and IOP controls among patients who have smoked in their lives.

Three different models (MD, VFI, and GPA) for visual field damage were used; nevertheless, there is no consensus about the best method.^36^ Smoking was significantly associated with VF deterioration in the three models studied. Other predictors for progression were IOP at diagnosis, VFI at recruitment, number of medications at the three-year follow-up, and SLT treatment. It is easy to understand IOP at diagnosis being a risk factor for visual field progression. Exfoliation glaucoma characterised by high IOP and increased IOP will damage ganglion cells and thus deteriorate the visual field. The other parameter that showed an association was the VFI values at recruitment. These parameters are directly related to the high IOP values at diagnosis. Patients with a more deteriorated visual field at diagnosis already had a loss of ganglion cells, which can explain the increased progression among these patients. The other parameters were the amount of SLT treatment and the number of medications. Patients showing faster progression were more often treated to slow down progression.

The three models for evaluating visual field progression differed in their nature. The advantage of the GPA model is that it is possible to obtain odds ratios (ORs) from the function (“Exp B”) and measure relative risks (RRs). In the case of the unadjusted model, the OR for smoking was 5.67, and the adjusted OR was 8.77. This OR is unrelated to the progression levels, as the variable is not continuous. The OR says that smokers had a five- to nine-fold increased risk of being placed among “progressors” compared with nonsmokers. The RR for smokers to develop progression was 1.95; this means that smokers are 95% more likely to develop visual field progression than nonsmokers. Even GPA seems an excellent method to analyse visual field damage as a binary variable, probably simplifying how the disease develops (linear). Finally, the three models complemented each other and added more information about disease progression.

A relationship between the number of cigarettes and progression was detected in the subgroup analysis. Patients who smoked more cigarettes showed increased progression. These results were shown in both the unadjusted (MD: *p* = 0.008; ROP: *p* = 0.006 and GPA: *p* = 0.04) and the adjusted (MD: *p* = 0.005; ROP: p ≤ 0.001 and GPA: *p* = 0.03) analyses in the three models studied. As the models were adjusted for IOP, visual field deterioration appeared unrelated to IOP. Nicotine induces vasoconstriction and decreased blood flow in the optic nerve; Mehra KS described this in 1976.^38^ These findings were described by several reports later on.^39,40^ Exfoliation glaucoma has been associated with an increased risk of vascular obliteration.^41,42^ An already-reduced blood flow in exfoliation glaucoma patients could be accentuated by nicotine use.

Another possible explanation for the results found could be genetic mechanisms. Exfoliation glaucoma has a solid genetic background; several genes have been studied. A previously published article^43^ showed an association between smoking and endothelial nitric oxide synthase (NOS3) gene variants in POAG patients. The results applied to both current and former smokers. The authors concluded that smoking could be a risk factor for developing POAG mediated through rs7830 NOS3. Exfoliation glaucoma has been associated with several genes. The most studied gene is the *LOXL1* gene.^44–47^ The hypothesis of genetic alterations in *LOXL1* due to smoking warrants further study.

The study has certain limitations. All included patients and their parents were born in Scandinavia. Therefore, the results from the present study cannot be applied to other populations. All patients were suffering from exfoliation glaucoma; the results cannot be applied to other types of glaucoma. Patients with significantly damaged visual fields were not included. This was to avoid “floor effects”, in which changes in the visual field cannot indicate whether the disease is progressing. The results from the study can be applied to early and moderate glaucoma.^37^ Another limitation is that only patients who smoked cigarettes were included. Other methods of nicotine intake were excluded. Another limitation of the study is that the majority of included patients were “former” smokers. The patients were consecutively included in our Ophthalmology Department. Even though this is a limitation (“former smokers”) and the results cannot be extrapolated to “current” smokers, the sample resembles our daily practice. Another limitation is that smoking status was studied through questionnaires that reflected baseline conditions; no information was collected about smoking status during the study. Another limitation of the study is that IOP was studied at the beginning and the end of the study; IOP changes during the study could influence the results. A similar applied to IOP treatment. It is possible that smokers were less likely to receive beta-blockers due to their potential side effects on the respiratory system. Further, behavioural factors have yet to be considered in the study. It is possible that smokers were less adherent to medications. Even other factors like body mass index (BMI) and alcohol consumption were not included in the study. Finally, no structural measurements of the optic nerve and/or parameters of ocular blood flow were evaluated in this study.

In conclusion, former smoking appears to be a risk factor for glaucoma progression in exfoliation glaucoma. Patients should be asked about smoking habits when the glaucoma diagnosis is established. Former smokers suffering from exfoliation glaucoma should be monitored often to detect the progression of the disease.

## Supplemental Material

sj-docx-1-ejo-10.1177_11206721241226990 - Supplemental material for Former smoking as a risk factor for visual field progression in exfoliation glaucoma patients in Sweden Supplemental material, sj-docx-1-ejo-10.1177_11206721241226990 for Former smoking as a risk factor for visual field progression in exfoliation glaucoma patients in Sweden by Marcelo Ayala in European Journal of Ophthalmology
