# Supplementary material for: Former smoking as a risk factor for visual field progression in exfoliation glaucoma patients in Sweden
Source: Eur J Ophthalmol. 2024 Jan 17;34(5):1481–8. doi: 10.1177/11206721241226990 (PMC11408981; doi:10.1177/11206721241226990)
Supplement: sj-docx-1-ejo-10.1177_11206721241226990 - Supplemental material for Former smoking as a risk factor for visual field progression in exfoliation glaucoma patients in Sweden [file sj-docx-1-ejo-10.1177_11206721241226990.docx]

Example of the questionnaire

Translation from the original questionnaire (in Swedish). The present is just a part of the initial questionnaire that includes several other questions than smoking.

Smoking

-Have you ever smoked more than 100 cigarettes in your life? Yes No

-Have you ever smoked ≥ once per week?

Yes No

If you answered “YES” to both questions, please answer the following:

-How many cigarettes you smoked per day?

-When you began smoking?

-When you finished smoking?

-Additional information.
